# Pregnancy's Stronghold on the Vaginal Microbiome

**DOI:** 10.1371/journal.pone.0098514

**Published:** 2014-06-04

**Authors:** Marina R. S. Walther-António, Patricio Jeraldo, Margret E. Berg Miller, Carl J. Yeoman, Karen E. Nelson, Brenda A. Wilson, Bryan A. White, Nicholas Chia, Douglas J. Creedon

**Affiliations:** 1 Department of Surgical Research, Mayo Clinic, Rochester, Minnesota, United States of America; 2 Institute for Genomic Biology, University of Illinois at Urbana-Champaign, Urbana, Illinois, United States of America; 3 Department of Animal and Range Sciences, Montana State University, Bozeman, Montana, United States of America; 4 Genomic Medicine Group, J. Craig Venter Institute, Rockville, Maryland, United States of America; 5 Department of Microbiology, University of Illinois at Urbana-Champaign, Urbana, Illinois, United States of America; 6 Animal Sciences, University of Illinois at Urbana-Champaign, Urbana, Illinois, United States of America; 7 Department of Physiology and Biomedical Engineering, Mayo College, Rochester, Minnesota, United States of America; 8 Obstetrics and Gynecology, Mayo Clinic, Rochester, Minnesota, United States of America; Argonne National Laboratory, United States of America

## Abstract

**Objective:**

To assess the vaginal microbiome throughout full-term uncomplicated pregnancy.

**Methods:**

Vaginal swabs were obtained from twelve pregnant women at 8-week intervals throughout their uncomplicated pregnancies. Patients with symptoms of vaginal infection or with recent antibiotic use were excluded. Swabs were obtained from the posterior fornix and cervix at 8–12, 17–21, 27–31, and 36–38 weeks of gestation. The microbial community was profiled using hypervariable tag sequencing of the V3–V5 region of the 16S rRNA gene, producing approximately 8 million reads on the Illumina MiSeq.

**Results:**

Samples were dominated by a single genus, *Lactobacillus*, and exhibited low species diversity. For a majority of the patients (n = 8), the vaginal microbiome was dominated by *Lactobacillus crispatus* throughout pregnancy. Two patients showed *Lactobacillus iners* dominance during the course of pregnancy, and two showed a shift between the first and second trimester from *L. crispatus* to *L. iners* dominance. In all of the samples only these two species were identified, and were found at an abundance of higher than 1% in this study. Comparative analyses also showed that the vaginal microbiome during pregnancy is characterized by a marked dominance of *Lactobacillus* species in both Caucasian and African-American subjects. In addition, our Caucasian subject population clustered by trimester and progressed towards a common attractor while African-American women clustered by subject instead and did not progress towards a common attractor.

**Conclusion:**

Our analyses indicate normal pregnancy is characterized by a microbiome that has low diversity and high stability. While *Lactobacillus* species strongly dominate the vaginal environment during pregnancy across the two studied ethnicities, observed differences between the longitudinal dynamics of the analyzed populations may contribute to divergent risk for pregnancy complications. This helps establish a baseline for investigating the role of the microbiome in complications of pregnancy such as preterm labor and preterm delivery.

## Introduction

The Human Microbiome Project (HMP) has helped define variations in the microbiome of healthy individuals and in providing a basis for assessing the role of the microbiome in disease [1]. Prior to HMP, our understanding of vaginal microbiota was based primarily on clinical diagnosis and culture techniques [2]. These do not always correlate to a particular microbiome and may be of limited use in guiding treatment [3]. With high-throughput sequencing, microbial community profiling [4] has allowed detailed resolution of the vaginal microbiome.

Gajer *et al.* [5] measured changes in microbial diversity and stability over time. Ecological diversity measures were consistent with past reports of low microbial diversity [6]–[8], however, a dynamic vaginal microbiome with marked changes through time was found. Given the stability and resilience manifested by multiple microbiome sites [9], the expectation for a healthy vaginal microbiome is one of stability and resilience as well. An unstable microbiome could be susceptible to invasion and dysbiosis.

Perturbations in the vaginal microbiome have been implicated in complications of pregnancy[10]–[15]. A study of the vaginal microbiome in pregnant and non-pregnant women has shown that pregnancy is characterized by a stable *Lactobacillus* dominated community [16], [17]. However, previous studies either did not examine time-longitudinal dynamics of the microbiome during pregnancy [16] or were restricted largely to particular minority groups [17]. Here, we provide evidence in support of *Lactobacillus* dominated microbiomes and higher stability during pregnancy in a time-longitudinal cohort that complements these existing studies.

## Materials and Methods

### Ethics Statement

Subjects were consented under IRB #10-006257, which was approved and reviewed by the Mayo Clinic Institutional Review Board. Written consent was provided by all subjects.

### Subject Enrollment

Here we report the results from 12 subjects enrolled at the Obstetric Division, Mayo Clinic, Rochester, MN under an IRB approval protocol (Vaginal Microbiome – Protocol 10-006257). The inclusion criteria were the following: ≥18 years of age; no known pregnancy complications at the first obstetrics visit (uncomplicated pregnancy); ability to provide written informed consent, willingness to participate in the mandatory translational research component of the study; and weight greater than 110 pounds (50 kilograms, a standard requirement in obstetrics studies that include blood draws). Patients having the following criteria were excluded from the study: known immunodeficiency; chronic, active viral infections, including HIV-1/2, HTLV-1/2, hepatitis B/C; known autoimmune disease, such as, rheumatoid arthritis or systemic lupus erythematosus; solid organ or transplant recipient; and multiple gestations. Upon enrollment the subjects were requested to fill a questionnaire about sexual and reproductive health and history. The metadata from the questionnaires was stored at REDCap [18]. Here we present the results from the first 12 patients with a healthy pregnancy progression and outcome.

### Sample Collection

**Figure 1:**
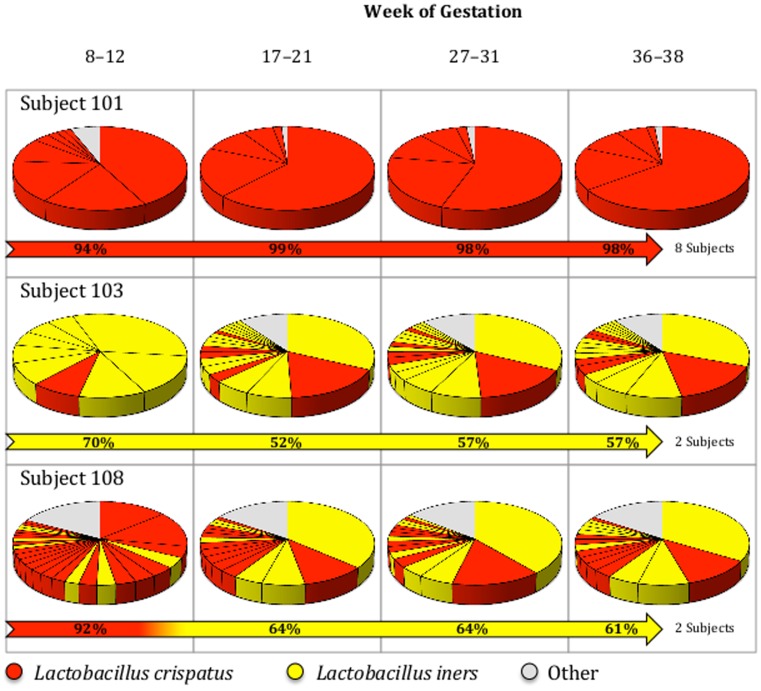
Pie charts representing the bacterial distribution found in each of the samples represented. Subject 101 is representative of 7 other patients with similar profiles (Subjects 104, 105, 106, 107, 109, 113 and 114). Subject 103 and Subject 110 have similar profiles. Subject 108 and Subject 111 have similar profiles. Divisions within the pie charts represent different OTUs (Operational Taxonomic Units – Strain level). “Other” represents OTUs with ≤1% abundance that belong to various species, excluding *L. crispatus* and *L. iners*.

Two Dacron swabs were used by the obstetrician or certified nurse midwife to sample the posterior fornix and cervix at time points: 8–12, 17–21, 27–31, and 36–38 weeks of gestation (Figure 1) and placed in a NAT (Nucleic Acid Transport, CentraCare Laboratory Services, St. Cloud, MN) collection tube. The last sample for one of the subjects (#110) was not collected. After collection the samples were kept at −80°C until processing to preserve the DNA material from degradation.

### Sample Processing

Once thawed the samples were centrifuged for 10 minutes at 10,000 *g* to collect the bacterial cells, and the supernatant was discarded. Genomic DNA extraction was performed by using the MoBio Ultraclean Soil Kit (MoBio Laboratories, Inc., Carlsbad, CA); however, instead of vortexing, MP FastPrep (MP Biomedicals, Solon, OH) for 40 seconds at 6.0 m/s was substituted to obtain a more effective and rapid lysis of the cells. Incubation period was done for a minimum of 30 minutes and a maximum of overnight. After extraction the DNA content was measured using High Sensitivity Qubit (Life Technologies Corporation, Carlsbad, CA) with the results ranging from 25–60 ng/µl. The V3–V5 region of the 16S rRNA was then amplified through a polymerase chain reaction (PCR) as follows: 25 µl of Kapa HiFi (Kapa Biosystems, Woburn, MA), 1.5 µl (10 uM) forward primer, 1.5 µl (10 uM) reverse primer, 50 ng of DNA with the remaining volume being added by molecular grade water (up to a final volume of 50 µl per reaction). The forward primer was the universal primer 357F (5′GTCCTACGGGAGGCAGCAG3′) with the added construct on the 5′ end of the 5′ Illumina Adapter (5′AATGATACGGCGACCACCGAGATCTACAC3′) + Forward Primer Pad (5′TATGGTAATT3′) to a total sequence: 5′AATGATACGGCGACCACCGAGATCTACACTATGGTAATTGTCCTACGGGAGGCAGCAG3′ and the universal bacterial reverse primer was 926R (5′CCGTCAATTCMTTTRAGT3′) with an added construct on the 5′ end of the reverse complement of 3′ Illumina adapter (5′CAAGCAGAAGACGGCATACGAGATGCCGCATTCGAT3′) + Barcode (12 base pairs) to a total sequence: 5′CAAGCAGAAGACGGCATACGAGATGCCGCATTCGATXXXXXXXXXXXXCCGTCAATTCMTTTRAGT3′. The barcode introduced in the reverse primer construct was unique to each sample, functioning as a genetic ID for sequencing. The PCR cycle was the following: 95°C for 3 min, 98°C for 20 sec, 70°C for 15 sec, 72°C for 15 sec, cycle repeated for 34 times and 72°C for 5 min. The products of the amplification were verified by TapeStation D1K Tape (2200 TapeStation Instrument, Agilent Technologies, Santa Clara, CA) to be free of contamination and the expected amplification size, approximately 700 base pairs. Upon verification the PCR products were purified using Agencourt AMPure (Beckman Coulter, Brea, CA). After purification the concentrations were measured using Qubit High Sensitivity and were found to be >1 ng/µl. The first samples 8–12 weeks of gestation were processed at the University of Illinois Urbana-Champaign with an identical protocol. Subsequently, all of the samples were then collected and sent to the University of Illinois Urbana-Champaign for 16S rRNA sequencing using a high-throughput next-generation Illumina MiSeq (250 PE, San Diego, CA) sequencing platform.

### Sequencing Outcome

A total of approximately 3,721,000 sequence reads (6,000 to 300,000 reads per sample) were obtained. After quality assessment (sequence reads with less than 187 bp in both R1 and R2 were discarded), 2,424,872 sequence reads (71 to 235,133 sequences per sample) remained and were found to be suitable for further analysis. Despite similar DNA concentrations the number of sequence reads was significantly different (lower) between the first collection time point and the last collection time point. For this reason these samples were repeatedly sequenced both at the University of Illinois Urbana-Champaign and the Mayo Clinic Medical Genome Center with similar results replicated. The reasons for the observed difference are unknown at this point, although they do not appear to have impacted the results.

### Sequence Analysis

Sequence reads were aligned with our own custom multiple alignment tool known as the Illinois-Mayo Taxon Operations for RNA Dataset Organization (IM-TORNADO) that merges paired end reads into a single multiple alignment and obtains taxa calls [19]. IM-TORNADO then clusters sequences into operational taxonomic units (OTUs) using AbundantOTU+ [20]. Further processing for visualization was performed using QIIME [21]. The sequence reads are publicly available in MG-RAST (http://metagenomics.anl.gov), sequence IDs: 4563804.3–4563899.3.

## Results

Our cohort consisted of 12 Caucasian subjects, 5 multiparous patients plus 7 primigravids who ranged in age from 24–36 years with a mean age of 29±4. The participants all self-reported having experienced regular periods prior to pregnancy and to be heterosexual. Within the cohort, there were no complications of pregnancy, including pre-term birth or gestational diabetes.

**Figure 2:**
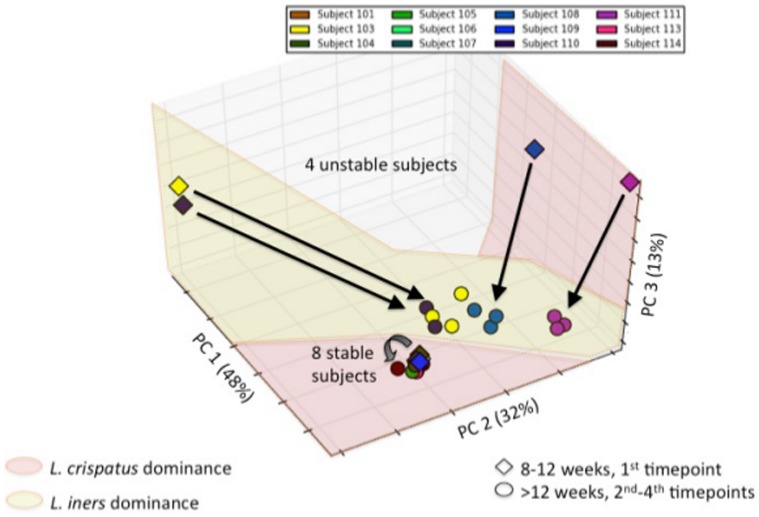
Principal Component Analysis (PCA) of temporal dynamics of the vaginal microbiome during pregnancy. Arrows indicate shifts from the first timepoint, taken at 8–12 weeks, and the subsequent timepoints. Notice that all temporal profiles that involve L. iners dominance involve a shift in the microbiome profile while purely *L. crispatus* dominated temporal profiles do not. Visualization carried out using Matplotlib version 1.2.1 (The MathWorks, Inc.).

The analysis of the sequence reads revealed that two species dominated the microbial content (>1% representation) of samples from the entire cohort (Table S2). The identified species were *L. crispatus* and *L. iners*. Among the 12 patients there were 3 profiles that could be distinguished (Figure 1): Eight of the subjects showed a high prevalence (>90%) of *L. crispatus* throughout pregnancy; two of the subjects showed a prevalence of *L. iners* (92–61%); and the remaining 2 subjects showed a transition in dominance after the first trimester of gestation from *L. crispatus* (70%) to *L. iners* (52–57%). Principal component analysis (PCA) confirmed the existence of the three profiles (Figure 2). Participants dominated by *L. iners* throughout pregnancy were significantly older (35±0 years old vs. 28±3 years old and 28±0 for the *L. crispatus* and transitory profiles, respectively; Table 1). All other metadata parameters were found not be significant (Metadata S1).

**Table 1:** Significant parameters found in the metadata.

Subject	Age (years)
**Crispatus**	
101	32
104	24
105	30
106	26
107	32
109	27
113	27
114	27
**Average**	**28**
**Standard Deviation**	3
**Iners**	
103	36
110	34
**Average**	35*
**Standard Deviation**	1
**Crispatus-Iners**	
108	28
111	28
**Average**	**28**
**Standard Deviation**	0

Significant differences from the *L. crispatus* profile, as determined by t-test, are highlighted by *.

**Figure 3:**
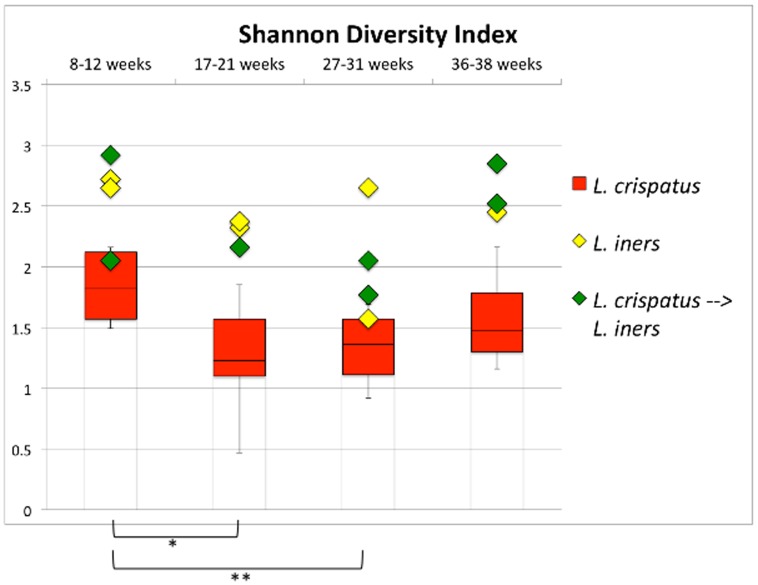
Shannon Diversity Index for the vaginal microbiome samples. The profile characterized by a dominance of *L. crispatus* throughout pregnancy (shared by 8 of the subjects) is significantly less diverse than the two other profiles (except during the first trimester). * p<0.05; ** p<0.005 on paired t-test.

Shannon's diversity indexes [22] (Figure 3) showed that the profile characterized by a dominance of *L. crispatus* throughout pregnancy is less diverse than the other profiles (except during the first trimester).

### Meta-Analysis

In order to allow for a better interpretation of the results we performed a meta-analyses comparing our results with those recently published by Romero *et al.* [17]. In this published work 22 pregnant women (19 African-American, 2 Caucasian and 1 Hispanic) were sampled throughout their pregnancy. Since all of our subjects were Caucasian, the comparison between both studies allows for an investigation of putative ethnical differences. It is well known that African-American healthy vaginal microbiotas exhibit a higher degree of inter-subject diversity than observed among Caucasian subjects [23]. Since the risk factor for the development of both gynecological [23] and obstetrical [24] morbidities is higher in African-Americans [23] it becomes of interest to characterize the healthy state of the vaginal microbiome in both groupings in order to investigate if the higher inter-subject variability is maintained in African-Americans or is homogenized by pregnancy.

**Figure 4:**
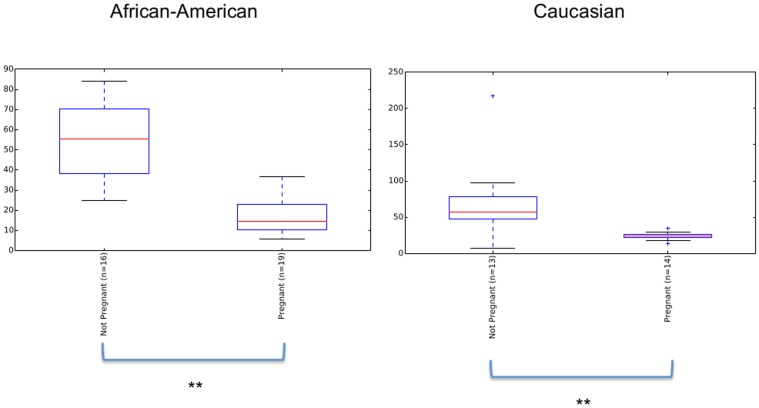
Pregnancy effect in African-American and Caucasian subjects as measured by Chao1 diversity Index. Diversity is significantly reduced during pregnancy in both ethnicities (**p<0.01, Monte Carlo analyses, 999 permutations).

**Figure 5:**
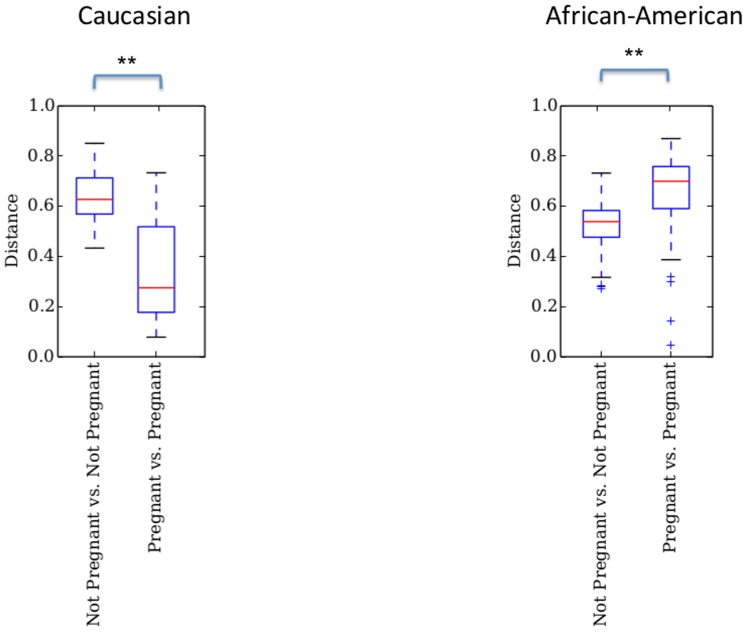
Unweighted Unifrac beta-diversity between pregnant and non-pregnant subjects within each ethnicity. Caucasians show significant convergence as a result of pregnancy (results confounded by platform effects – See Table S1) while African-Americans show significant divergence in pregnancy when compared to non-pregnant subjects. **p<0.001, Monte Carlo analyses, 999 permutations.

**Figure 6:**
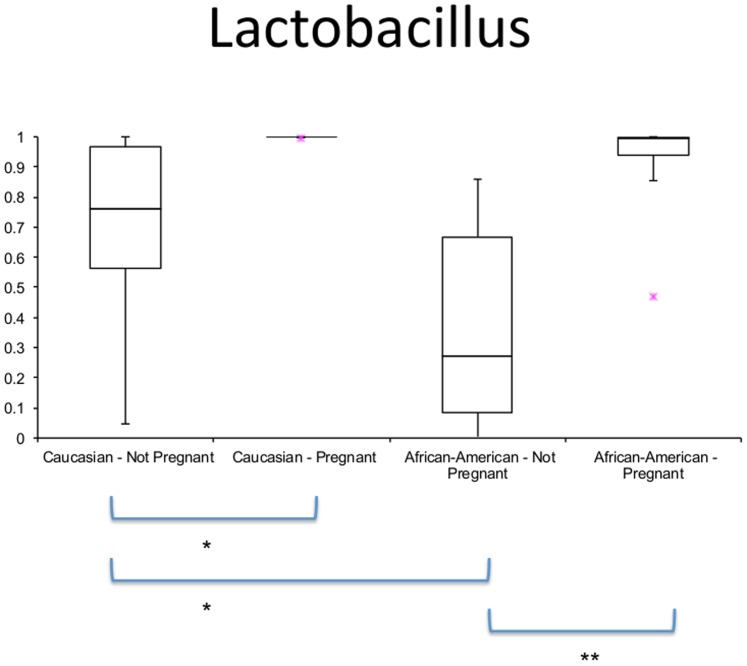
*Lactobacillus* species frequencies across ethnicities and pregnancy states. Significant differences were found between all categories except between pregnant Caucasians and pregnant African-Americans (*p<0.05;**p<0.01; Monte Carlo analysis, 999 permutations).

We subjected data from Romero *et al* [17] to our analysis, which focuses on the microbial community dynamics between trimesters, in order to compare results with this concurrent study. Due to the different 16S rRNA region amplified in the different studies (V1–V3 in Romero's study and V3–V5 in this study) and the different sequencing platforms (454 in Romero's study and Illumina in this study) the comparison is limited (See Methods S1 for procedures and limitations; Figure S1; Figure S2, and Table S1 for the parameters analyzed). Despite limitations due to the different 16s rRNA primers and sequencing platform utilized in the Romero *et al*. study, some meaningful comparative analyses were possible through the use of reference OTUs [21]. The results show that in both ethnicities pregnancy significantly decreases α-diversity (Figure 4). Comparison in overall diversity between ethnicities did not reveal any significant differences regardless of pregnancy state (Figure S3). Overall, we found divergence between subjects to be higher between non-pregnant subjects in Caucasians than between pregnant ones (p<0.01, Figure 5). The opposite was observed between non-pregnant and pregnant African-Americans, where inter-subject variability was lower than between pregnant individuals (p<0.01, Figure 5). Comparison of the relative abundance of the genus *Lactobacillus* in the non-pregnant state is significantly higher in Caucasians than African-Americans but this proportion becomes non-significant when pregnant subjects are compared (Figure 6).

**Figure 7:**
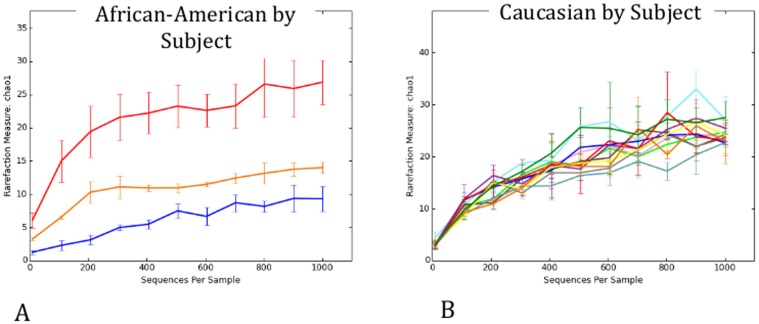
Estimation of species richness according to Chao 1 index. A – African-American subjects (Red – N009, Orange – N018, Blue – N017). Diversity between subjects is significant and therefore does not allow for the grouping of the subjects for analytical purposes. B – Caucasian subjects. No significant differences in diversity were found among subjects.

**Figure 8:**
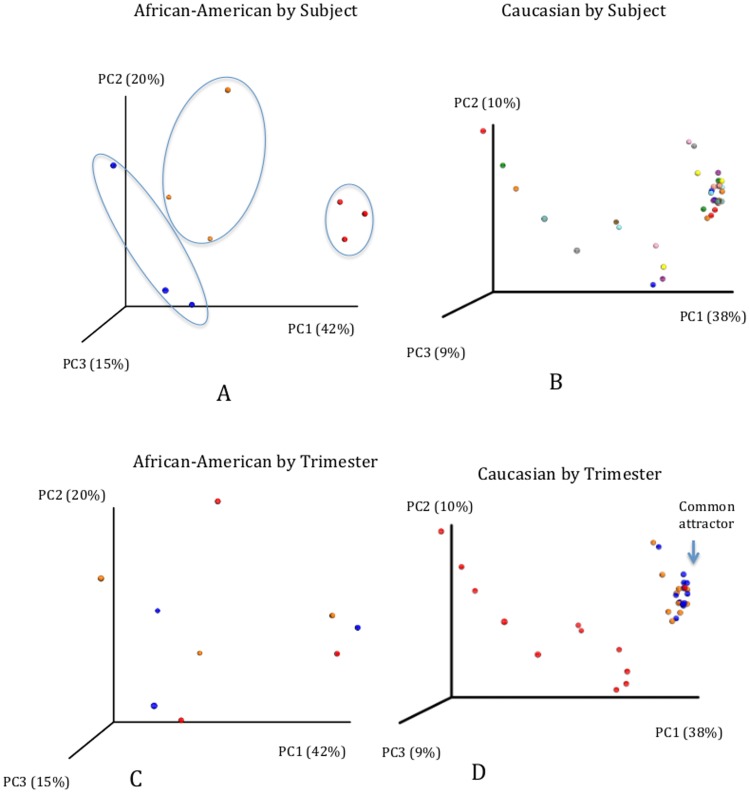
Unweighted PCA analysis by Subject and Trimester. A – African-American by Subject. Clustering by Subject can be observed. B – Caucasian by Subject. No clear clustering is observed. C – African-American by Trimester. No common attractor can be identified. D – Caucasian by Trimester. A common attractor can be observed. Plots C and D – 1^st^ Trimester – Red; 2^nd^ Trimester – Blue; 3^rd^ Trimester – Orange.

While many meaningful results arose from our meta-analysis, certain examinations were unfortunately obscured by the effect of different sequencing platforms. (Table S1; Figure S2). For the pregnant subjects, the effects of ethnicity on β-diversity appear to be overwhelmed by the effects of primer choice and platform. A major limiting factor was present in side-by-side time-longitudinal analyses and ethnicity as we identified only 3 African-American subjects with longitudinal data across all 3 trimesters (all other subjects did not have samples collected during the first trimester). Though small in size, we nonethless compared the results from these 3 African-American subjects and our 12 Caucasian subjects and showed that the diversity observed between the African-American subjects varied widely across these 3 cases in comparison to the 12 Caucasian subjects (Figure 7). The PCA plots shown in Figure 8 further showed that the African-American subjects clustered by subject and did not show a common attractor as the pregnancies progressed. This is in contrast with what was observed in the Caucasian subjects, which did not cluster by subject, but instead showed a strong common attractor towards the later stages of the pregnancy.

## Discussion

The vaginal microbiome seems unique among the sites sampled for the HMP in terms of its low diversity [1], [8], [16]. A recent cross-sectional study by Aagaard *et al.* [16] found enrichment for *Lactobacilli* in the vaginal microbiome of pregnant women. Our results are consistent with this finding and identify only two species that comprised >1% of the microbial population in our samples—*L. crispatus* and *L. iners*. Furthermore, our longitudinal sampling reveals that this low diversity is also matched by a high stability across the course of pregnancy. This contrasts with non-pregnant women who have been shown to exhibit fluctuations with menstrual cycles [5].

Numerous factors may contribute to the stability of the vaginal microbiome during pregnancy, including: a lack of cyclic fluctuations in hormones, absence of menstrual flow, absence of alterations in the cervical or vaginal secretions associated with the reproductive cycle, or changes in sexual activity during pregnancy [25], [26]–[30]. Some or all of these factors could be implicated in the stability of the vaginal microbiome during pregnancy [31].

The Shannon Diversity Index revealed that the *L. crispatus*-dominant profile was associated with a lower diversity, suggesting that *L. crispatus* is more exclusionary when dominant than *L. iners*. Our findings are consistent with those reported in a cross-sectional study by Ravel *et al.* [8] that profiled the vaginal microbiome in non-pregnant women. The relative amount of dominance exhibited by *L.*crispatus and *L.*iners may be a factor in the susceptibility to changes in the microbiota. In addition, *L.iners* has been identified as a transitional vaginal microbe, potentially indicating susceptibility to dysbiosis or a recovery from it [32]. In this study we did not verify any difference in the pregnancy progression or outcome of women whose vaginal microflora was dominated by *L.iners*, although the fact that these profiles were more diverse than the ones dominated by *L.crispatus* may indicate a higher susceptibility to change. Overall, it is well known that *Lactobacillus* species actively protect themselves and the vaginal environment from invaders by the production of lactic acid which acidifies the vaginal pH as well as the production of H_2_O_2_ which prevents ascending infection [33]. At least 25% of pre-term births are associated with infection, and a healthy vaginal microbiome may be a stronghold against potential microbial invaders [34].

One of the limitations of this study was the small sample size and the homogeneous population. All women in this study were Caucasian. However, the large majority of participants in the complementary study from Romero *et al*. [17] are from African-American women. Although still limited by numbers, our meta-analysis and comparison allowed us to draw inferences in these two populations. Perhaps, the most important limiting factor in the meta-analysis are the differences in hypervariable 16S rRNA regions amplified (V1-V2 in Romero's dataset and V3-V5 in our study) and sequencing platforms used (454 in Romero's and Illumina in ours). Attempts to single out the biases and artifacts introduced by these factors were made and the result of such comparisons determined that these artifacts made many of the direct comparisons between both datasets inconclusive (details presented in the Methods S1, Figure S2 and Table S1). This further highlights the importance of standards in the burgeoning field of microbiome research [35] in the women's reproductive tract.

Despite some of these technical challenges important conclusions can be inferred from the results. It is well known that there are differences between the microbiotas of African-American and Caucasian women, with the latter being more often reported to have a higher representation of *Lactobacillus* species [23]. The vaginal health benefits of a *Lactobacillus* dominated flora are well-described, and the fact that African-American women are more susceptible to gynecological morbidities such as bacterial vaginosis and vaginitis may be linked to a flora depleted in *Lactobacillus* [23] or a functional substitute that will maintain the acidity and peroxide contents that shield the vaginal canal from a multitude of pathogens. The dominance of *Lactobacillus* in Caucasian women seen on our cohort is telling when coupled with the fact that pregnancy complications leading to preterm labor and delivery are higher in African-American women, at a rate of 16–18% of all deliveries, when compared to 5–9% in Caucasian [24].

A significant difference was found in the dynamics of the vaginal microbiome where Caucasians experienced a diminished divergence of the microbiota as pregnancy progressed, while African-American subjects exhibited an augmented divergence between subjects. Ravel et al. [8] have shown that African-American subjects are more likely to have a vaginal microbiome not dominated by *Lactobacillus*. However, Zhou et al. [23] have shown that Caucasians vaginal microbiota is commonly dominated by similar numbers of more than one species of *Lactobacillus* while this was rarely observed in African-Americans. Hence, both these observations confound diversity estimations between the two ethnic groups. We have not found a statistically significant difference in the vaginal microbiome diversity between African-Americans and Caucasians either non-pregnant or pregnant (Figure S3). What we did find was that the non-pregnant state is significantly more diverse than the pregnant state regardless of ethnicity (Figure 4). In accordance with Romero et al. [23] we verified that the proportion of *Lactobacillus* dominated communities is different between the two ethnicities when not pregnant (higher in Caucasians) but becomes statistically indistinguishable during pregnancy (Figure 6). This finding is an indicator that pregnancy has a marked effect in the vaginal microbiome, which experiences a complete dominance by *Lactobacillus* species regardless of ethnicity.

Interestingly, examination of the microbial community dynamics using principal coordinate analysis reveals that Caucasian women cluster by trimester towards a common attractor (Figure 8), suggesting that these subjects share a common microbiome dynamic. On the other hand, African-American women cluster by subject and do not show a common attractor (Figure 8). This is also supported by the wider analysis shown on Figure 5. It is tempting to speculate that these differences in microbial dynamics may underlie the increased risk of pregnancy complications in particular individuals in the African-American population. However, due to the multitude of other factors that may also vary across these two populations, it is difficult to isolate the cause. Nonetheless, it is reasonable to consider the role of the microbiome in preterm birth and other pregnancy complications. This study highlights the importance of larger studies comparing subjects with different ethnical backgrounds that also analyze specific pregnancy complications such as preterm labor.

## Supporting Information

Figure S1 **PCA showing the clustering by sequencing platform.**
(TIF)

Figure S2 **Venn diagram of samples analyzed.**
(TIF)

Figure S3 **Chao diversity index in non pregnant and pregnant subjects (results confounded by the platform effect in pregnant subjects - see Table S1).** No significance was found between the two groups of subjects (p>0.05, Monte Carlo analysis, 999 permutations).
(TIF)

Table S1 (PDF)

Table S2 **Supplemental OTU Table.**
(XLSX)

Metadata S1 (CSV)

Methods S1 (DOCX)
