# Supplementary figures and images for: Pregnancy's Stronghold on the Vaginal Microbiome
Source: PLoS One. 2014 Jun 4;9(6):e98514. doi: 10.1371/journal.pone.0098514 (PMC4045671; doi:10.1371/journal.pone.0098514)

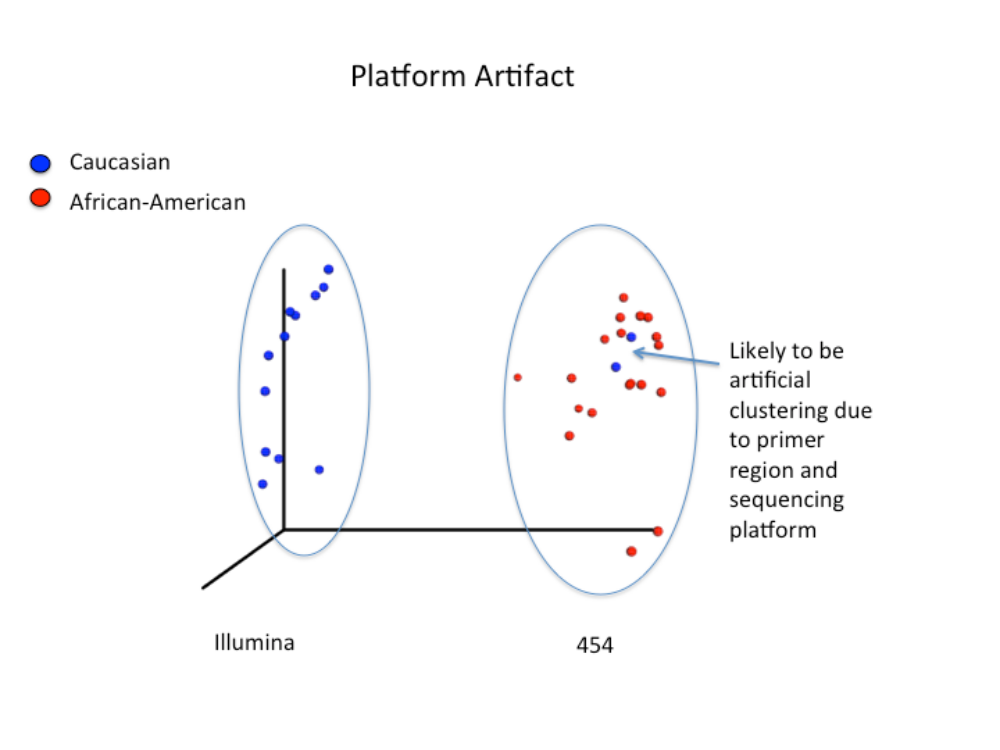

Supplement: Figure S1 — PCA showing the clustering by sequencing platform. (TIF) [file pone.0098514.s001.tif]

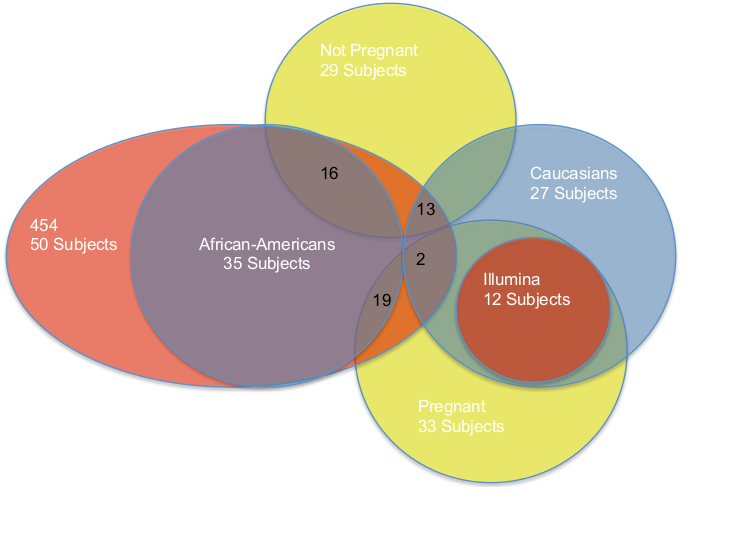

Supplement: Figure S2 — Venn diagram of samples analyzed. (TIF) [file pone.0098514.s002.tif]

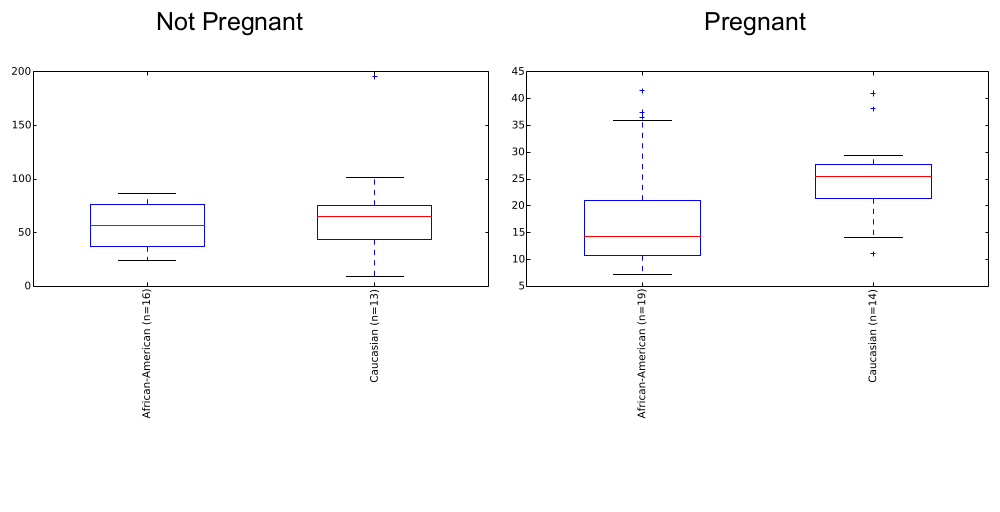

Supplement: Figure S3 — Chao diversity index in non pregnant and pregnant subjects (results confounded by the platform effect in pregnant subjects - see Table S1). No significance was found between the two groups of subjects (p>0.05, Monte Carlo analysis, 999 permutations). (TIF) [file pone.0098514.s003.tif]
