# Supplementary material for: Pregnancy's Stronghold on the Vaginal Microbiome
Source: PLoS One. 2014 Jun 4;9(6):e98514. doi: 10.1371/journal.pone.0098514 (PMC4045671; doi:10.1371/journal.pone.0098514)
Supplement: Table S1 — (PDF) [file pone.0098514.s004.pdf]

Supplemental Table 1 – Comparison between main parameters. The number of subjects in prohibitive for most of the analyses that would allow the disentanglement of the variables.

Unweighted Beta-Diversity - Monte Carlo 999 permutations	
Overall Comparison	p-value
454 (50 subjects) vs Illumina (12 subjects)	**0.001**
African-American (35 subjects) vs Caucasian (27 subjects)	0.224
Pregnant (33 subjects) vs No Pregnant (29 subjects)	**0.001**
**Platform Effect**	
**454** & African-American & Pregnant (19 subjects) vs **Illumina** & African-American & Pregnant **(0 subjects)**	NA
**454** & Caucasian & Pregnant **(2 subjects)** vs **Illumina** & Caucasian & Pregnant (12 subjects)	NA
**454** & African-American & Not Pregnant (16 subjects) vs **Illumina** & African-American & Not Pregnant **(0 subjects)**	NA
**454** & Caucasian & Not Pregnant (13 subjects) vs **Illumina** & Caucasian & Not Pregnant **(0 subjects)**	NA
**Ethnicity Effect**	
**African-Americа**n & 454 & Pregnant (19 subjects) vs **Caucasian** & 454 & Pregnant **(2 subjects)**	NA
**African-American** & Illumina & Pregnant **(0 subjects)** vs **Caucasian** & Illumina & Pregnant (12 subjects)	NA
**African-American** & 454 & Not Pregnant (16 subjects) vs **Caucasian** & 454 & Not Pregnant (13 subjects)	**0.001**
**African-American** & Illumina & Not Pregnant **(0 subjects)** vs **Caucasian** & Illumina & Not Pregnant **(0 subjects)**	NA
**Pregnancy Effect**	
**Pregnant** & 454 & African-American (19 subjects) vs **Not Pregnant** & 454 & African-American (16 subjects)	**0.001**
**Pregnant** & Illumina & African-American **(0 subjects)** vs **Not Pregnant** & Illumina & African-American **(0 subjects)**	NA
**Pregnant** & 454 & Caucasian **(2 subjects)** vs **Not Pregnant** & 454 & Caucasian (13 subjects)	NA
**Pregnant** & Ilumina & Caucasian (12 subjects) vs Not **Pregnant** & Illumina & Caucasian **(0 subjects)**	NA
