# Supplementary material for: Pregnancy's Stronghold on the Vaginal Microbiome
Source: PLoS One. 2014 Jun 4;9(6):e98514. doi: 10.1371/journal.pone.0098514 (PMC4045671; doi:10.1371/journal.pone.0098514)
Supplement: Methods S1 — (DOCX) [file pone.0098514.s007.docx]

Supplemental Methods

Comparative Meta-Analysis

Datasets Processing

Sequence data generated through the 454 platform was retrieved from the SRA database. The sequence reads were then processed through the UPARSE pipeline as described in Edgar, 2013. Our dataset (generated through Illumina platform) was processed through IM-Tornado (as described in the article). Both datasets were then combined and QIIME was used to generate a closed-reference OTU.

Combination of Datasets

The 2 datasets were originated in different laboratories with different sample processing protocols, different amplification primers and different sequencing platforms (454 and Illumina). This results in potentially multiple sources impacting variation between both datasets. In order to allow for a comparison, both datasets were combined and a closed reference (GreenGenes) OTU (Operational Taxonomic Unit) was used for the assembly of common OTUs.

After the assembly, the resulting phylogenetic tree and taxonomy table were processed through QIIME with a cutoff of 1,000 sequences per sample, to allow for an even sampling. Due to the use of a closed-reference OTU, the taxonomic assignments lacked an adequate resolution at the species level. Hence, inferences from the results in the meta-analysis section of the paper were based on genus assignments.

The effect of the platform was analyzed by comparing pregnant Caucasian women (12 Illumina subjects and two 454 subjects). The comparison between both platforms was only possible in this circumstance because of the lack of matching comparable sets. The limited comparison showed a very high likelihood of strong platform and/or primer effects, with the two 454 Caucasian subjects being the only ones clustering with African-American subjects (Supplemental Figure 1). Hence, the direct comparison of the datasets was deemed inconclusive leading to their separate analysis (Supplemental Figure 2 and Supplemental Table 1). The analysis was limited to within ethnicity variation and dynamics throughout time. All analyses were performed using QIIME v.1.8.0.

**Supplemental Table 1.** Comparison between main parameters. The number of subjects is prohibitive for most of the analyses that would allow the disentanglement of the variables.

| Unweighted Beta-Diversity - Monte Carlo 999 permutations |  |
| --- | --- |
| Overall Comparison | p-value |
| 454 (50 subjects) vs Illumina (12 subjects) | **0.001** |
| African-American (35 subjects) vs Caucasian (27 subjects) | 0.224 |
| Pregnant (33 subjects) vs No Pregnant (29 subjects) | **0.001** |
| **Platform Effect** |  |
| **454** & African-American & Pregnant (19 subjects) vs **Illumina** & African-American & Pregnant **(0 subjects)** | NA |
| **454** & Caucasian & Pregnant **(2 subjects)** vs **Illumina** & Caucasian & Pregnant (12 subjects) | NA |
| **454** & African-American & Not Pregnant (16 subjects) vs **Illumina** & African-American & Not Pregnant **(0 subjects)** | NA |
| **454** & Caucasian & Not Pregnant (13 subjects) vs **Illumina** & Caucasian & Not Pregnant **(0 subjects)** | NA |
| **Ethnicity Effect** |  |
| **African-America**n & 454 & Pregnant (19 subjects) vs **Caucasian** & 454 & Pregnant **(2 subjects)** | NA |
| **African-American** & Illumina & Pregnant **(0 subjects)** vs **Caucasian** & Illumina & Pregnant (12 subjects) | NA |
| **African-American** & 454 & Not Pregnant (16 subjects) vs **Caucasian** & 454 & Not Pregnant (13 subjects) | **0.001** |
| **African-American** & Illumina & Not Pregnant **(0 subjects)** vs **Caucasian** & Illumina & Not Pregnant **(0 subjects)** | NA |
| **Pregnancy Effect** |  |
| **Pregnant** & 454 & African-American (19 subjects) vs **Not Pregnant** & 454 & African-American (16 subjects) | **0.001** |
| **Pregnant** & Illumina & African-American **(0 subjects)** vs **Not Pregnant** & Illumina & African-American **(0 subjects)** | NA |
| **Pregnant** & 454 & Caucasian **(2 subjects)** vs **Not Pregnant** & 454 & Caucasian (13 subjects) | NA |
| **Pregnant** & Ilumina & Caucasian (12 subjects) vs Not **Pregnant** & Illumina & Caucasian **(0 subjects)** | NA |

References:

Edgar RC. 2013. UPARSE: highly accurate OTU sequences from microbial amplicon reads. Nature Methods. 10: 996-998.
